# Reflections on the Teaching Symposium at the Microbiology Society Annual Conference 2023

**DOI:** 10.1099/acmi.0.000676.v3

**Published:** 2023-09-22

**Authors:** Melissa M. Lacey, Kirsty L. Jones, Monika Gostić, Victoria Easton, Georgios Efthimiou, Bridget G. Kelly, Alison I. Graham

**Affiliations:** ^1^​ Department of Biosciences and Chemistry, Sheffield Hallam University, Sheffield, UK; ^2^​ Department of Biological Sciences, School of Health, Science and Wellbeing, Staffordshire University, Stoke-on-Trent, UK; ^3^​ School for Medicine, Medical Sciences and Nutrition, Aberdeen University, Aberdeen, UK; ^4^​ School of Molecular and Cellular Biology, University of Leeds, Leeds, UK; ^5^​ Centre for Biomedicine, Hull York Medical School, Hull, UK; ^6^​ Department of Life and Health Science, Dundalk Institute of Technology, Dundalk, Ireland; ^7^​ School of Medicine, Newcastle University, Newcastle-upon-Tyne, UK

**Keywords:** active learning, education, inclusive practice, outreach, pedagogy, public engagement

## Abstract

The Microbiology Society Education and Outreach Network (EON) recently hosted the Teaching Symposium at the Microbiology Society Annual Conference, sponsored by *Access Microbiology*. The presence of the Symposium as an established parallel session within the wider Annual Conference reflects the importance of high-quality, contemporary microbiology education and outreach delivered in an enthusiastic and inclusive manner. At the 2023 Symposium, a variety of pedagogical research projects in higher education learning, teaching and assessment, as well as public engagement projects, were showcased through invited talks, offered talks, flash talks and posters. The event was attended by up to 70 delegates. Several themes were noted throughout the day: engaging with Gen Z (Generation Z, those born between 1996 and 2010), active learning, art in science and engaging with non-higher education (HE) audiences. Inclusivity was a key driver in the organization of the Symposium; the room was set up to encourage discussion and participants could ask questions using an online platform as well as speaking in the room. We now encourage all speakers to consider publishing their work as a peer-reviewed article for further dissemination and impact.

## Data Summary

No data were generated during this research or are required for the work to be reproduced.

## Introduction

The Education and Outreach Network (EON) is made up of national and international Microbiology Society members interested in educating students at all stages, as well as members of the public, about microbiology. It supports all Society members to share ideas and best practice and to connect with other educators. The Network has expanded recently to around 12 members and two co-Chairs who represent a range of career stages from PhD students to established academic colleagues with interests and experience in education and public engagement research [1–5].

EON was proud to host the Teaching Symposium at the Microbiology Society Annual Conference, sponsored by *Access Microbiology*. The Teaching Symposium started in 2018 as a pre-conference satellite session and has since become embedded as a parallel session within the main conference, reflecting the importance of high-quality, contemporary microbiology education and outreach.

The 2023 Symposium included a mix of invited talks, offered talks, flash talks and posters [6], with up to 70 delegates attending, some staying for the whole day and some dropping in for specific talks. Attendance remained consistent throughout the day. We have a strong drive to ensure the Teaching Symposium is welcoming and inclusive, a core strand running from planning to execution. This article will provide a snapshot of the overarching themes and highlights of the Symposium, as well as reflections by those who organized and chaired the sessions.

## Overarching themes of the Symposium

### Engaging with Gen Z

Gen Z (Generation Z, those born between 1996 and 2010) are accustomed to high-quality, bite-sized content, both in education and on social media. Tina Joshi [7] opened the Symposium by stressing the importance of talking to students and engaging in a way that feels authentic to them (which, for Tina, included bringing *Love Island* into discussions on potential disease transmission!). This ethos of making content relevant to the audience to retain their engagement continued into the public engagement and science communication talks of the afternoon. Alison Cottell discussed her epidemiology teaching practices, which allowed students to critically engage in the topic after living through a pandemic, despite the ‘epidemiology fatigue’ that could bring. She advocated the use of authentic assessments, such as debunking fake news in Instagram posts.

Mel Lacey gave a glimpse into how Gen Z students are using social media for discussions around their teaching and assessment. The vast majority of the students who responded to their questionnaire use one of the top three platforms (Snapchat, WhatsApp and Facebook) to discuss their studies at least once per week. Bridget Kelly described how she encourages students to collaborate on difficult topics by embedding problem-based learning [8] in undergraduate immunology teaching.

### Active learning

Many of the speakers in the morning session embedded active learning [9] in their practice. Nicholas Harmer, building on previous work [10], demonstrated how a range of different approaches can be used to provide students with individual datasets to create authentic and robust assessments, and, crucially, how automated marking can be designed in parallel. Relatively common programmes such as Excel and R can be used to create these individual data sets for exams and coursework. In contrast, Gemma Wattret showcased more specialized employability software, Shortlist Me, to capture digital interviews to increase awareness of commercial and career skills amongst students. Jerry Reen emphasized the benefits of repetition and multiple modalities by initially teaching students how to construct representations of plasmids with physical jigsaws before moving on to using virtual reality.

Game-based learning [11], a theme in last year’s Symposium, came through strongly again this year. David Negus won an *Access Microbiology* award for the poster that supported his talk about a board game to illustrate the antibiotic development pipeline. Students designed elements of their boards to represent the challenges in developing new antibiotics and demonstrate how money is so easily spent during antibiotic development. A public engagement escape room presented by Maitreyi Shivkumar, showing antiviral drug discovery, was not only a great example of science outreach but would also make a fantastic teaching laboratory session.

### Art in science

**Fig. 1.**
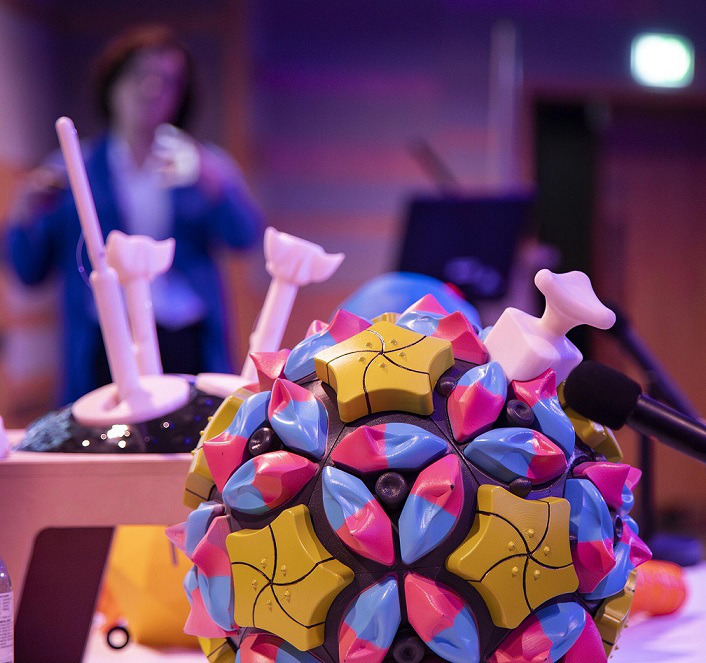
Briony Thomas presenting her collaborative research on art in science with tactile models in the foreground.

The powerful nature of partnerships and collaborations outside microbiology to create impactful public engagement was seen across the afternoon session. Joanna Verran shared a collaborative, citizen science project centred around kombucha. This project brought together colleagues from the worlds of film, fashion, food and fermentation. Chloe James further exemplified the power of multidisciplinary collaboration when sharing her experiences of working with artists to create an immersive installation of video, sound and sculpture. Briony Thomas and Morgan Herod brought together designers and scientists who worked with students to co-create physical and virtual virus models (Fig. 1). These presentations showed how exploring science through art can result in both beautiful and effective public engagement [12].

### Engaging with non-higher education (HE) audiences

**Fig. 2.**
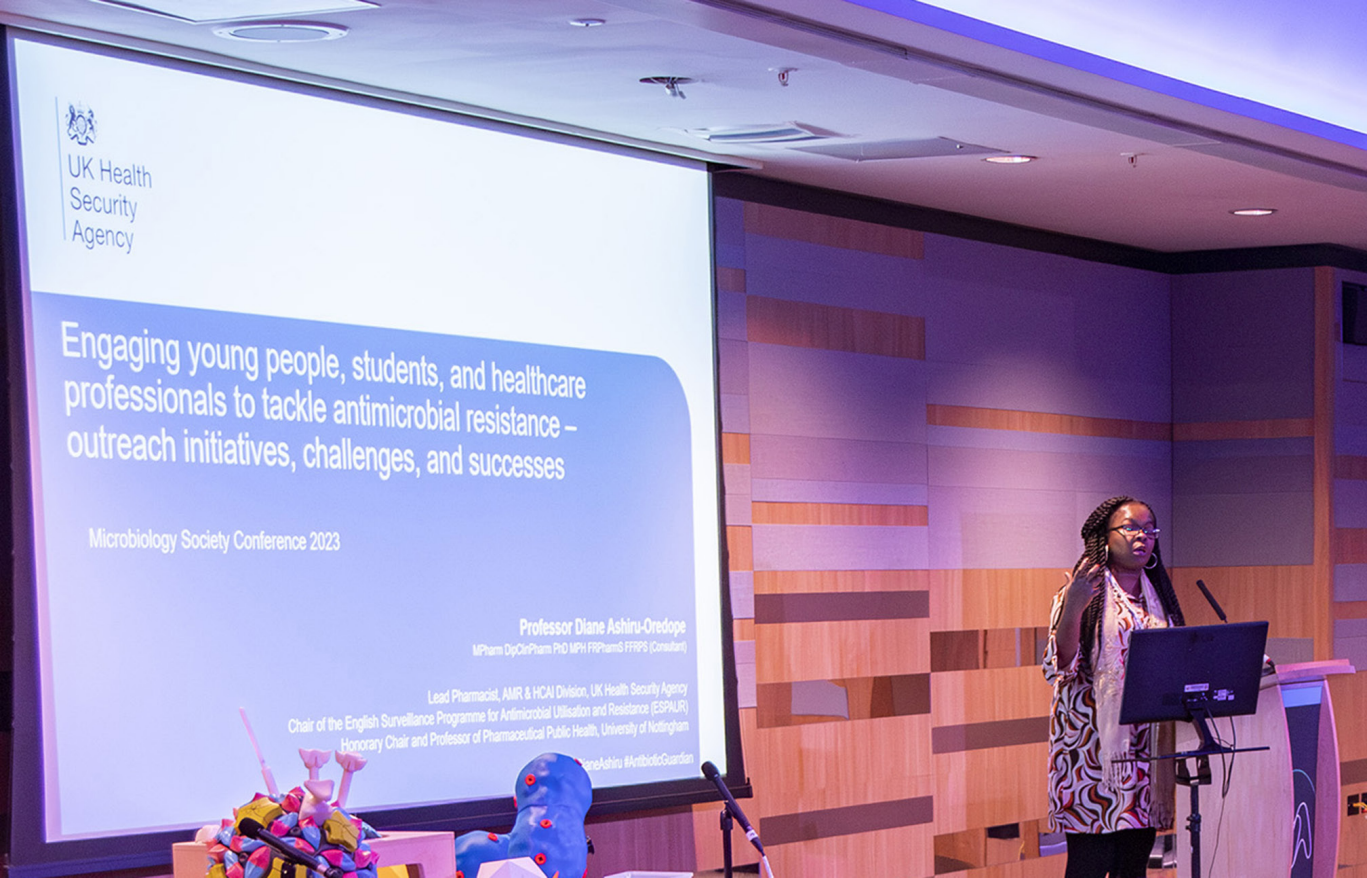
Diane Ashiru-Oredope sharing a range of methods to engage both HE and non-HE audiences.

Linda Oyama shared her remarkable experiences in reaching over 2600 primary school children in Northern Ireland, largely during the pandemic, to bring them authentic outreach activities. This is highly commendable even without considering the additional steps that were needed to comply with the restrictions in place at the time. Diane Ashiru-Oredope shared a range of public engagement resources that spanned primary school children to healthcare workers (Fig. 2). Two particular highlights were e-Bug, an online repository of free activities to engage children and young people with antibiotic resistance [13], and the Antibiotic Guardian scheme [14], which invites members of the public and professionals to pledge an action to make better use of antibiotics. The importance of including students as presenters to increase audience diversity at a blended art and science event was demonstrated by Kelly Capper-Parkin [15], whose supporting poster won an *Access Microbiology* award.

## Reflections on the Symposium

This article, and the following reflections, are written by those who organized and chaired sessions at the Teaching Symposium. A core aim of the Teaching Symposium was to create a friendly, inclusive and interdisciplinary space. We hoped that delegates would be inspired by the presentations and posters, see themselves as part of a community of practice, and have the opportunity to network and ‘find their people’.

**Fig. 3.**
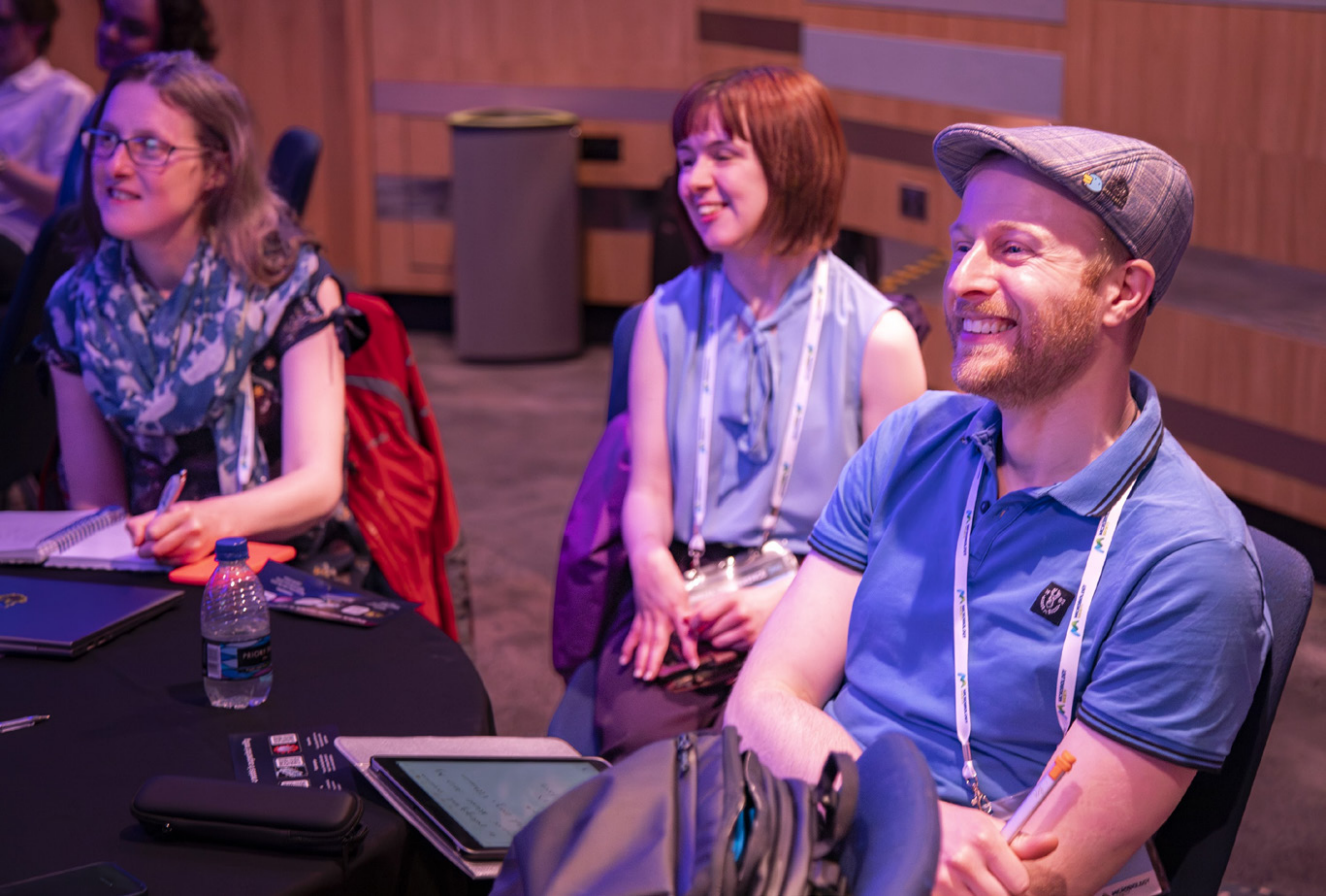
Cabaret-style seating throughout the Symposium facilitated discussion.

Inclusivity was a key driver for many decisions taken regarding the Symposium (just as it would be for an effective teaching session [16]). We were conscious that participants can sometimes find asking questions in front of a large group daunting. To ease the pressure of public speaking, we implemented an anonymous online platform https://padlet.com/ (Padlet) [17] for collating questions that were read out by the Session Chair, as well as taking questions in the room. To further encourage interaction and discussion, the room was set up with cabaret-style seating, which is based on a horseshoe-style seating arrangement (Fig. 3) [18].

The programme of the Symposium was designed to cover a range of contributions, from small-scale classroom interventions to large multi-person collaborative outreach ventures. Presentations ranged from 30 min presentations from invited speakers to 5 min flash talks that allowed a number of contributors to showcase their good practice. Some speakers gave slide-based presentations, whereas others chose more demonstration-based formats. On reflection, the variety of timings and formats helped to keep engagement high throughout the day. In particular, including flash talks allowed a wider range of speakers to contribute than would otherwise have been possible. Although each talk was short, delegates were seen to follow up with speakers at break times and we saw contact details being noted to follow up after the Symposium. Delegates were asked to feed back at the end of the day and the variety of activities covered was mentioned as a highlight by several participants.

Many participants remarked on the friendliness of the Symposium, which had created a relaxed environment. Speakers were enthusiastic and passionate, not just about their topics, but also in the way they communicated. This enthusiasm transferred to the audience, with many thought-provoking questions being asked both in-person and online. The final session asked delegates to record their biggest inspiration of the day. Many were impressed by how science and arts can come together to achieve high impact. Delegates also reported that they would appreciate help in implementing new ideas in their teaching and outreach. EON is actively working with the Microbiology Society to curate existing teaching and outreach resources on their website to make these more accessible.

A secondary aim of the Symposium was to increase the visibility and accessibility of pedagogy and outreach research within the microbiology community. The Symposium was sponsored by *Access Microbiology* and the session organizers felt this sponsorship gave an implied pathway to publication. Throughout the poster sessions, Society journals highlighted research that aligned with their scope. *Access Microbiology* tagged appropriate and relevant posters with a ‘We like your work – submit to *Access Microbiology*’ sticker. As well as this tangible intervention, all speakers at the Symposium were invited by the Pedagogy Editor to consider submitting their research as a peer-reviewed publication to *Access Microbiology*. We felt that encouraging presenters to publish their work was well received. This was reflected in the range of responses to a discussion around publication, with some presenters already having submitted work, some aiming to prepare manuscripts and others having not yet considered the impact of their work past their presentation or poster.

Overall, there was a clear message from delegates that they would like to see the Symposium retained as a parallel session next year. We are pleased that what started as a satellite to the main conference has become thoroughly embedded alongside the scientific sessions. The authors feel this is important because it allows delegates whose primary focus is not education to dip in and out of the Symposium whilst also providing a community of practice for those who are more education- and outreach- focused. It is our hope that the Teaching Symposium will go from strength to strength, providing a supportive, practice-based forum for education and outreach in a microbiology setting.

## References

[1] Duckett CJ, Hargreaves KE, Rawson KM, Allen KE, Forbes S (2021). Nights at the museum: integrated arts and microbiology public engagement events enhance understanding of science whilst increasing community diversity and inclusion. Access Microbiol.

[2] Efthimiou G, Tucker NP (2020). Microbes against humanity, a workshop game for horrible students: using a creative card game in higher education microbiology teaching. Access Microbiol.

[3] Graham AI, Harner C, Marsham S (2022). Can assessment-specific marking criteria and electronic comment libraries increase student engagement with assessment and feedback?. Assess Eval High Educ.

[4] Pincott H, Hughes M, Cummins T, Morse DJ (2023). Building blocks of biofilms - an engaging and hands-on microbiology outreach activity for school children and the general public. Access Microbiol.

[5] Lacey MM, Shaw H, Abbott N, Dalton CJ, Smith DP (2022). How students’ inspirations and aspirations impact motivation and engagement in the first year of study. Educ Sci.

[6] Microbiology Society website (2023). Annual conference programme. https://microbiologysociety.org/event/annual-conference/annual-conference-2023.html.

[7] Joshi LT (2021). Using alternative teaching and learning approaches to deliver clinical microbiology during the COVID-19 pandemic. FEMS Microbiol Lett.

[8] Bledsoe KE (2011). Managing problem-based learning in large lecture sections. Biosci Educ.

[9] Theobald EJ, Hill MJ, Tran E, Agrawal S, Arroyo EN (2020). Active learning narrows achievement gaps for underrepresented students in undergraduate science, technology, engineering, and math. Proc Natl Acad Sci U S A.

[10] Harmer NJ, Hill AM (2021). Unique data sets and bespoke laboratory videos: teaching and assessing of experimental methods and data analysis in a pandemic. J Chem Educ.

[11] Gao F, Li L, Sun Y (2020). A systematic review of mobile game-based learning in STEM education. Educ Tech Res Dev.

[12] Braund M, Reiss MJ (2019). The ‘Great Divide’: how the arts contribute to science and science education. Can J Sci Math Techn Educ.

[13] E-Bug website (2023). https://www.e-bug.eu.

[14] Antibiotic Guardian website (2023). https://antibioticguardian.com.

[15] Lacey M, Capper-Parkin K, Schwartz-Narbonne R, Hargreaves K, Higham C (2023). University student-led public engagement event: increasing audience diversity and impact in a non-science space. Access Microbiol.

[16] Dewsbury B, Brame CJ (2019). Inclusive teaching. CBE Life Sci Educ.

[17] Padlet https://padlet.com/.

[18] McCorskey JC, McVetta RW (1978). Classroom seating arrangements: Instructional communication theory versus student preferences. Comm Educ.

